# The evolving role of regulatory T cells in pulmonary diseases: immunomodulatory mechanisms and translational directions revealed by bibliometric analysis

**DOI:** 10.3389/fmed.2026.1839305

**Published:** 2026-06-09

**Authors:** Qiuxia Wang, Jianfeng Jiang, Huici Yao, Ying Zhu

**Affiliations:** Department of Pediatrics, The Affiliated Hospital of Jiangsu University, Zhenjiang, China

**Keywords:** bibliometric analysis, cytokines, immunomodulation, lung diseases, regulatory T cells

## Abstract

**Background:**

Regulatory T cells (Tregs) play important roles in immune homeostasis and pulmonary disease progression. However, the global research landscape, thematic evolution, and emerging frontiers of Treg research in pulmonary diseases remain unclear. This study aimed to systematically characterize research trends and knowledge structures in this field using bibliometric methods.

**Methods:**

A total of 9,079 deduplicated publications related to Tregs in pulmonary diseases were retrieved from the Web of Science Core Collection and Scopus databases from 2000 to 2025. Bibliometric and visualization analyses were performed to evaluate publication trends, country contributions, collaboration networks, keyword co-occurrence, co-citation patterns, and emerging hotspots.

**Results:**

The field exhibited a tri-phasic growth pattern: a slow initial phase (2000–2008), a rapid growth phase (2009–2019), and the current high-output phase (2020–present). The United States played a dominant role in the early period, while China has emerged as the top-producing country annually since 2021. Keyword co-occurrence and co-citation analyses revealed a thematic shift from foundational immune regulation and cytokine networks toward emerging translational topics, including immunotherapy, the tumor microenvironment, microbiota-associated immune regulation, and chronic inflammatory lung diseases.The analyses also identified the most prominent keywords, clusters, and highly cited references that together define the evolving knowledge structure of Treg research in pulmonary diseases.

**Conclusion:**

This bibliometric analysis provides a comprehensive overview of global trends and emerging frontiers in Treg research in pulmonary diseases. Emerging therapeutic strategies suggested in the literature, including targeting Tregs, the tumor microenvironment, or the gut microbiota, may guide future translational research and precision immunotherapy development in pulmonary diseases.

## 1 Introduction

The lung is a vital organ for gas exchange and is highly susceptible to a variety of external insults, including infections, toxins, and environmental irritants. Consequently, a broad spectrum of pulmonary diseases may develop, encompassing infectious, immune-related, chronic inflammatory, and neoplastic disorders, such as pneumonia, bronchial asthma, chronic obstructive pulmonary disease (COPD), and lung cancer (1). In recent years, accumulating evidence has demonstrated that the immune system plays a critical role in maintaining pulmonary homeostasis as well as in the initiation and progression of lung diseases. In particular, dysregulation of adaptive immunity has been closely associated with unfavorable outcomes in multiple pulmonary disorders (2). Therefore, elucidating the underlying mechanisms of immune regulation is of considerable importance for the prevention and treatment of lung diseases.

Regulatory T cells (Tregs) were first identified in 1995, and their key transcription factor, Forkhead box P3 (Foxp3), was subsequently characterized in 2001. Tregs, typically characterized by the CD4 + CD25 + Foxp3 + phenotype, represent a specialized subset of CD4 + T cells with essential immunosuppressive and immune homeostatic functions (3). Previous studies have shown that Tregs are involved not only in tissue homeostasis and injury repair but also in immune regulation across a wide range of diseases, including autoimmune disorders, inflammatory diseases, and cancer (4). In lung tissue, Tregs contribute to the maintenance of pulmonary immune balance by regulating the proliferation and differentiation of alveolar epithelial cells and microvascular endothelial cells (5). Under pathological conditions, however, dysregulation of Treg function has been closely associated with various pulmonary diseases. For example, an imbalance between Tregs and Th17 cells has been reported in patients with asthma and COPD (6), whereas significantly reduced numbers of Tregs have been observed in idiopathic pulmonary fibrosis (IPF) (7). In non-small cell lung cancer (NSCLC), increased Treg infiltration has been associated with immunosuppression and poor prognosis (8). With the rapid growth of related studies, research on Tregs in lung diseases has gradually evolved into multiple interconnected research hotspots and emerging directions.

Bibliometrics is a quantitative analytical approach that applies mathematical and statistical methods to academic publications and is widely used to identify research hotspots, knowledge structures, and developmental trends within a specific field. Compared with traditional narrative reviews, bibliometric analysis enables the systematic exploration of collaborative relationships and evolutionary patterns among countries, institutions, authors, and keywords based on large-scale bibliographic databases, while also visualizing emerging frontiers and shifts in research focus (9). Given that research on Tregs in lung diseases spans multiple disciplines, including immunology, respiratory medicine, and oncology, and is characterized by rapidly expanding and highly heterogeneous research topics, bibliometric methods are particularly suitable for systematically mapping the developmental trajectory of this field, identifying core research themes, and predicting future research directions. However, to date, a comprehensive bibliometric analysis focusing specifically on Tregs in lung diseases remains lacking.

Therefore, the present study retrieved publications related to Tregs and lung diseases published between January 1, 2000 and December 31, 2025 from the Web of Science Core Collection (WoSCC) and Scopus databases, and conducted a visualized bibliometric analysis using CiteSpace. By analyzing publication trends, country and institutional collaboration networks, influential authors, keyword clusters, and burst terms, this study aimed to systematically reveal the research hotspots, knowledge structure, and potential future directions in this field, thereby providing references for subsequent basic and clinical research.

## 2 Materials and methods

### 2.1 Data sources and search strategy

The bibliographic data used in this study were retrieved from the Web of Science Core Collection (WoSCC) and Scopus databases, which are widely recognized as authoritative and comprehensive sources for bibliometric analyses. To minimize potential bias caused by continuous database updates, all literature retrieval and data downloads were completed on a single day, January 22, 2026. The retrieval period covered publications from January 1, 2000, to December 31, 2025, thereby ensuring complete inclusion of the 2025 annual publication data.

The search strategy was developed by combining controlled vocabulary terms and free-text keywords related to regulatory T cells and pulmonary diseases using Boolean operators. Searches were restricted to the title, abstract, and keyword fields. The detailed retrieval strategies for WoSCC and Scopus are provided in Supplementary material 1.

The search formulas were as follows:

*WoSCC*: TS = ((“regulatory T cell*” OR “Treg*” OR “regulatory T lymphocyte*” OR “Foxp3 + T cell*” OR “CD4 + CD25 + Foxp3+”) AND (“lung disease*” OR “pulmonary disease*” OR “respiratory disease*” OR “lung disorder*” OR “pulmonary disorder*” OR “lung injury*” OR “pulmonary fibrosis” OR “asthma” OR “COPD” OR “lung cancer”)).

*Scopus*: TITLE-ABS-KEY ((“regulatory T cell*” OR “Treg*” OR “regulatory T lymphocyte*” OR “Foxp3 + T cell*” OR “CD4 + CD25 + Foxp3+”) AND (“lung disease*” OR “pulmonary disease*” OR “respiratory disease*” OR “lung disorder*” OR “pulmonary disorder*” OR “lung injury*” OR “pulmonary fibrosis” OR “asthma” OR “COPD” OR “lung cancer”)).

Only peer-reviewed articles and reviews published in English were included. Editorial materials, letters, conference abstracts, meeting proceedings, corrections, notes, book chapters, early access documents, and duplicated records were excluded. All records were exported in plain text format with full records and cited references. The bibliographic records retrieved from WoSCC and Scopus were merged using NoteExpress software (version 4.3.0).

**Figure 1:**
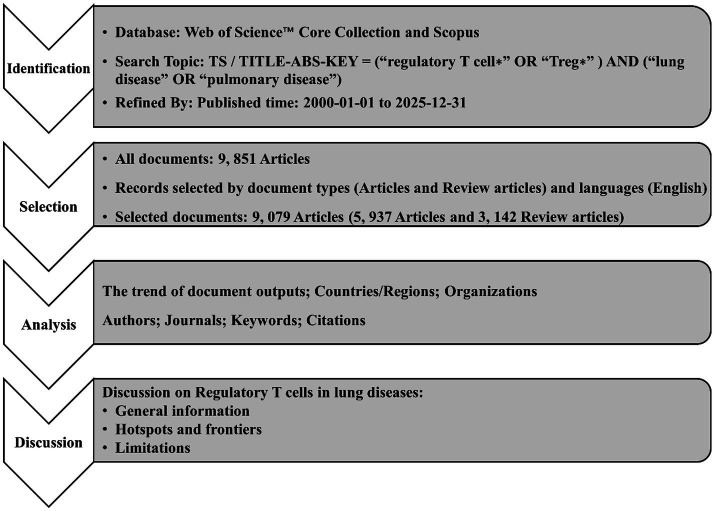
Flowchart of the document-screening process and research framework.

To construct a non-redundant dataset, a multi-step deduplication process was performed. First, duplicate records were identified through exact matching of Digital Object Identifiers (DOIs). For records without DOI information, duplicate screening was subsequently conducted based on combinations of article title, first author, publication year, and source journal, followed by manual verification to ensure deduplication accuracy. After removal of duplicate records, the final bibliographic dataset was obtained, and full records with cited references were exported in plain text format for subsequent bibliometric analyses. In cases of inconsistent metadata or incomplete records after database merging, records from the Web of Science Core Collection (WoSCC) were preferentially retained because they provide more comprehensive citation information and better compatibility with CiteSpace, particularly for co-citation analysis and other core bibliometric functions. The detailed literature screening and selection process is illustrated in Figure 1.

### 2.2 Bibliometric analysis and visualization

Bibliometric analysis and scientific knowledge mapping were performed using CiteSpace (version 6.4. R1 Advanced), a widely used bibliometric visualization software developed by Chaomei Chen for detecting emerging trends and knowledge structures in scientific literature. The analysis mainly included annual publication outputs, countries/regions, institutions, authors, journals, keywords, cited references, and collaboration networks. The parameter settings in CiteSpace were configured as follows: the time slicing was set from 2000 to 2025, with 1 year per slice; the selection criteria used the g-index with a scaling factor k = 25; node types included country, institution, author, cited author, cited journal, keyword, and cited reference. Pathfinder and pruning sliced networks were applied to simplify the network structure and improve visualization clarity.

In the visualization maps generated by CiteSpace, nodes represented bibliometric entities such as authors, institutions, or keywords, whereas links between nodes represented collaborative, co-occurrence, or co-citation relationships. The size of each node reflected the frequency or citation counts of the corresponding item. Betweenness centrality was used to evaluate the importance of nodes within the collaboration network, and nodes with centrality values greater than 0.10 were considered pivotal points connecting different research domains. Keyword co-occurrence analysis, clustering analysis, timeline visualization, and citation burst detection were further performed to identify research hotspots, thematic evolution, and emerging trends in Treg-related pulmonary disease research. Citation burst analysis was conducted to detect references or keywords receiving rapidly increasing attention within a specific period, thereby identifying frontier topics and potential future directions. The quality and reliability of clustering results were evaluated using modularity Q and weighted mean silhouette S values. Generally, Q > 0.3 indicates a significant clustering structure, whereas S > 0.7 suggests high clustering consistency and reliability.

### 2.3 Statistical analysis

Descriptive statistical analyses were performed to summarize annual publication outputs and citation trends. Microsoft Excel 2024 was used for data organization and basic statistical analyses. Graphs and visual knowledge maps generated by CiteSpace were further optimized for presentation quality.

The study was conducted based on publicly available bibliographic data; therefore, ethical approval and informed consent were not required.

## 3 Results

### 3.1 The trend of document outputs

On January 22, 2026, a total of 9,079 publications were collected from the Web of Science™ Core Collection and Scopus databases after merging and removing duplicates. This corpus consisted of 5,937 research articles and 3,142 review articles. The literature screening process and analytical framework are presented in Figure 1.

**Figure 2:**
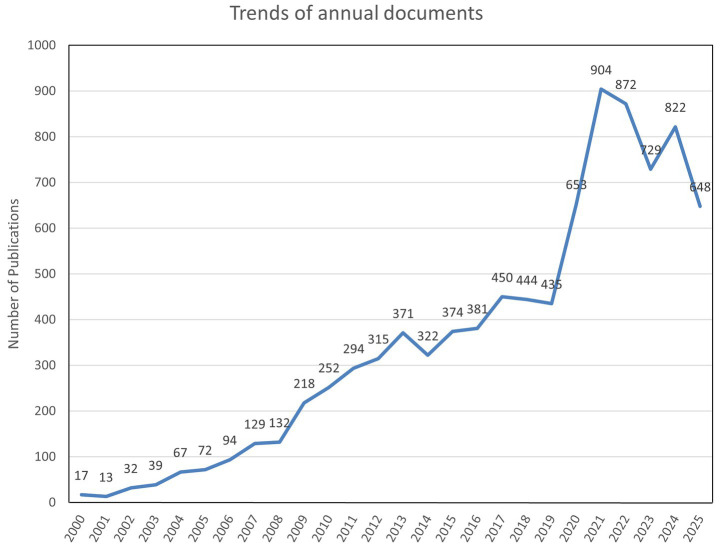
Trends of annual documents related to Tregs in lung diseases.

As shown in Figure 2, the research trend can be divided into three general phases. In the first phase, from 2000 to 2008, the annual output grew gradually from a low baseline, with the lowest point in 2001 (only 13 relevant publications) and a slow year-on-year increase until 2008. The second phase, spanning 2009 to 2019, witnessed a rapid rise in the annual number of publications on Tregs in lung diseases, despite a slight fluctuation in 2014. The third phase, from 2020 to the present, is characterized by a consistently high annual output with minor variations, peaking at 904 publications in 2021. In 2025, the output remained substantial with 648 publications, indicating that research on Tregs in pulmonary diseases has stabilized at a high level in recent years. These results demonstrate that since 2009, investigating the role of Tregs in lung diseases has become a broadly and continually focused research hotspot.

### 3.2 Countries/Regions

In CiteSpace, each node represents a country or region, and the radius of the node is proportional to its contribution to research on Tregs in lung diseases. Links between nodes indicate collaborative relationships between countries/regions, with thicker lines representing stronger collaboration intensity. Node centrality reflects the extent of connectivity with other nodes and is proportional to the size of the surrounding purple ring; a larger purple ring indicates higher centrality.

**Figure 3:**
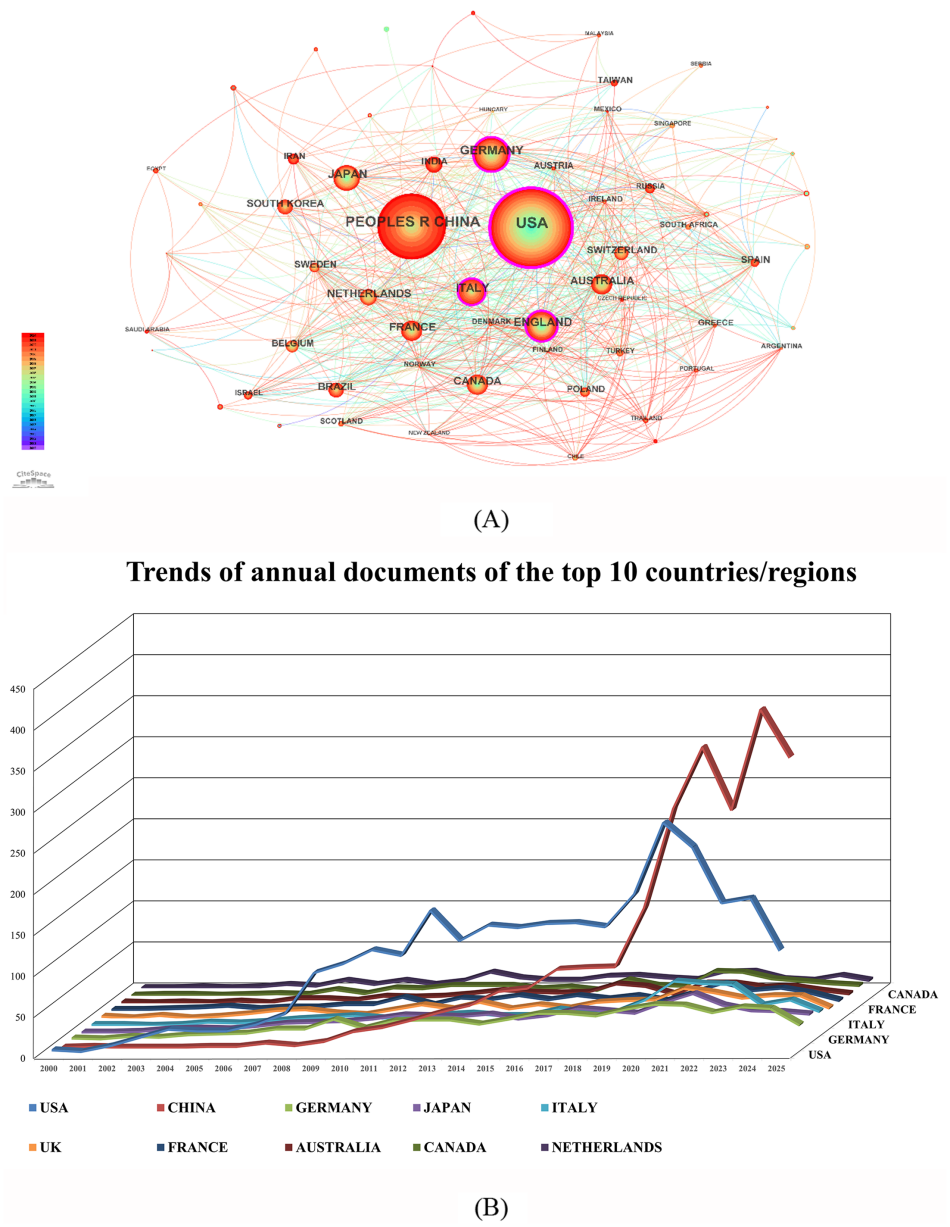
**(A)** Visualization map of countries/regions collaboration analysis. **(B)** Trends of annual documents of the top 10 countries/regions. The distance between two circles represents their coauthorship relationship, while the thickness of the connecting line indicates the strength of the relationship.

From 2000 to 2025, a total of 112 countries/regions participated in research related to Tregs in lung diseases (Figure 3A). The country/region collaboration network demonstrated extensive international cooperation across the field. The United States, China, and Germany ranked as the top three countries in terms of publication output. Among them, the United States not only produced a large number of publications but also exhibited the highest total link strength (TLS) and relatively high centrality, indicating its prominent position within the international collaboration network. The top 10 countries/regions are presented in Figure 3B. Temporal trend analysis showed that the United States was among the earliest countries to engage in this research field and maintained a leading publication output prior to 2020. Since 2021, China has surpassed other countries in annual publication volume, becoming the most productive country in this field. In addition, the United States, the United Kingdom, and Germany exhibited relatively high centrality values within the collaboration network, suggesting that these countries play important bridging roles in international academic cooperation.

### 3.3 Organizations

**Figure 4:**
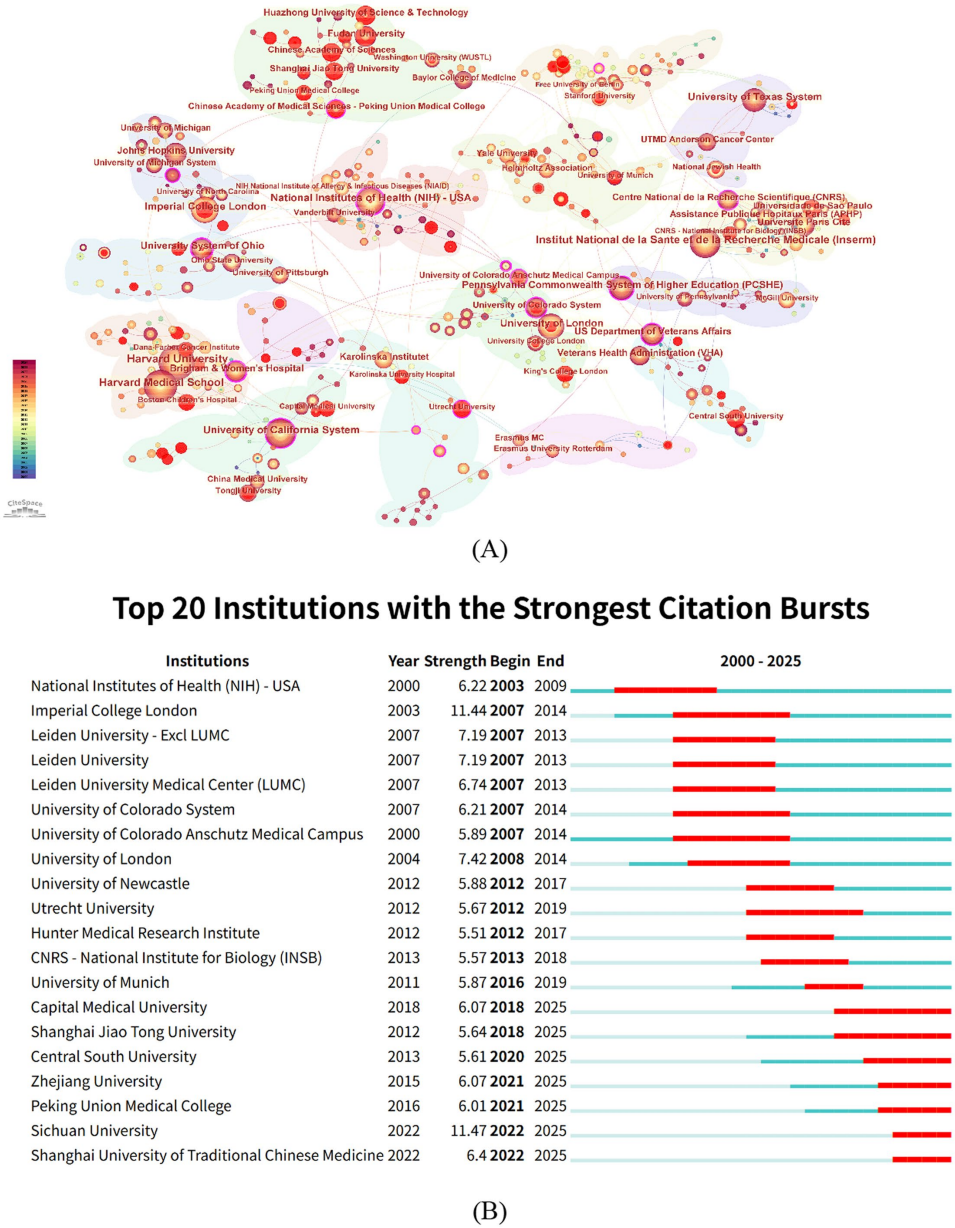
**(A)** Visualization map of organizations collaboration analysis. **(B)** Top 20 organizations with the strongest citation bursts.

A total of 540 institutions were included in the institutional collaboration network analysis and visualized using CiteSpace (Figure 4A). To improve the readability of the network map and reduce interference from low-frequency nodes, a minimum publication threshold of 20 publications per institution was applied, based on commonly adopted practices in bibliometric studies and the distribution characteristics of institutional publication output in the present dataset. Accordingly, only institutions with ≥20 publications were displayed and labeled in the network map. This threshold enabled preservation of the major collaborative structure while avoiding excessive network complexity caused by an overabundance of nodes.

In the collaboration network, each node represents an institution, and node size is positively correlated with publication output. Links between nodes indicate collaborative relationships between institutions. Nodes with high centrality are highlighted by purple outer rings, indicating their important bridging roles within the collaboration network. The results showed that Harvard Medical School ranked first in publication output, whereas Brigham and Women’s Hospital exhibited the highest centrality value (Table 1), suggesting its important role in institutional collaboration within this field. Citation burst analysis at the institutional level demonstrated that Sichuan University showed the strongest burst intensity. Temporal trend analysis further indicated a gradual increase in research activity among Chinese institutions in recent years. Institutions including Sichuan University, Fudan University, and Shanghai Jiao Tong University exhibited notable citation bursts after 2018 (Figure 4B). Several institutions maintained burst activity through 2025, indicating sustained academic attention in the field of Treg research in lung diseases.

**Table 1:** Top 10 institutions on research of Tregs in lung diseases.

Rank	Institution	Count	Institution	Centrality
1	Harvard Medical School	160	Brigham & Women’s Hospital	0.31
2	Ministry of Education of the People’s Republic of China	148	University System of Ohio	0.30
3	Inserm	131	Johns Hopkins Medicine	0.24
4	National Institutes of Health (NIH)	119	National Institutes of Health (NIH)	0.23
5	Fudan University	109	Cleveland Clinic Foundation	0.19
6	Chinese Academy of Medical Sciences & Peking Union Medical College	107	Philipps University Marburg	0.16
7	Huazhong University of Science and Technology	98	University of California System	0.15
8	Universidade de São Paulo	93	Medical University of Vienna	0.15
9	The University of Texas MD Anderson Cancer Center	92	Chinese Academy of Medical Sciences - Peking Union Medical College	0.13
10	Imperial College London	90	Pennsylvania Commonwealth System of Higher Education	0.12

### 3.4 Authors

**Figure 5:**
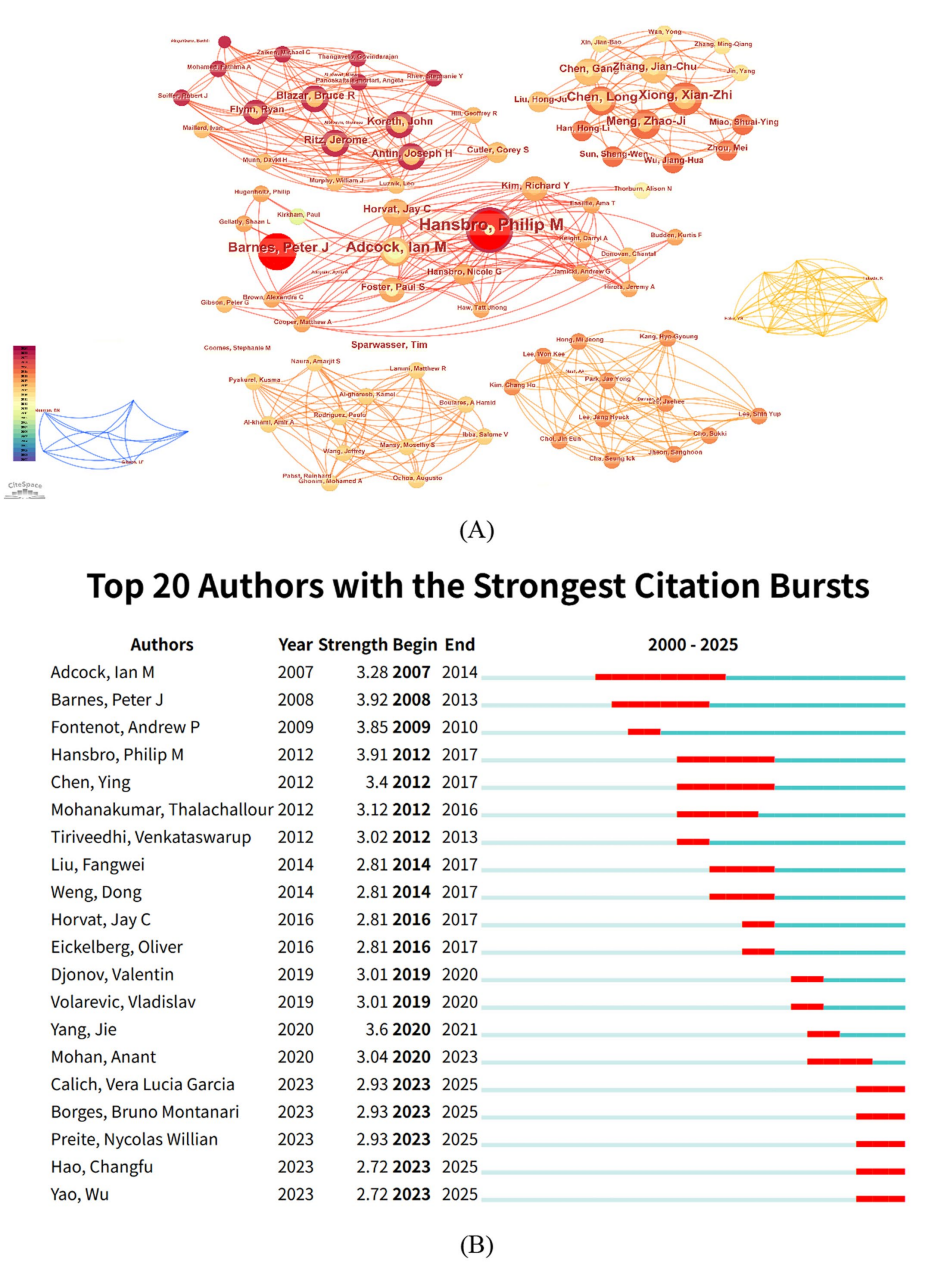
**(A)** Visualization map of authors collaboration analysis. **(B)** Top 20 authors with the strongest citation bursts.

After excluding isolated nodes, a total of 1,027 authors were included in the author collaboration network analysis (Figure 5A). To improve the readability of the network map, CiteSpace automatically generated several collaborative clusters based on co-authorship relationships. Links between nodes represent collaborations among authors, and node size is positively correlated with publication output. The results showed that Bart N Lambrecht from Ghent University had the highest number of publications in this field, whereas Philip M Hansbro from The University of Newcastle exhibited the highest centrality value, indicating a strong bridging role within the author collaboration network. Author co-citation analysis demonstrated that Shimon Sakaguchi from Osaka University was the most frequently co-cited author (Figure 5B). In addition, Peter J Barnes from Imperial College London showed the strongest citation burst intensity, indicating that his publications received substantial attention during a specific period. Temporal trend analysis further revealed increasing research activity among Chinese scholars in recent years. Authors such as Ying Chen and Jie Yang exhibited citation bursts that persisted through 2025, suggesting sustained academic interest in their recent research contributions.

### 3.5 Journals

**Figure 6:**
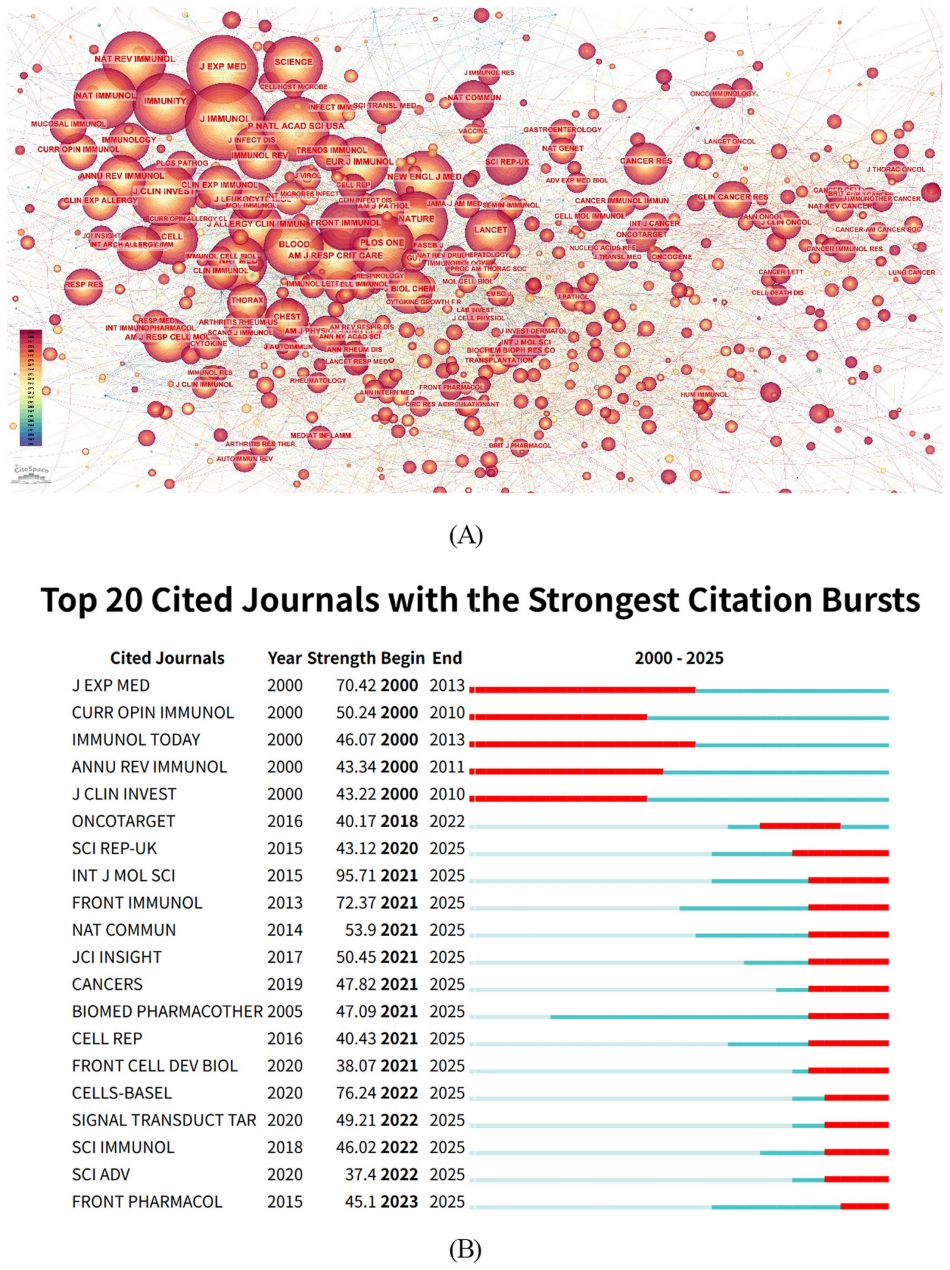
**(A)** Analysis of collaborative network visualization of journal citations. **(B)** Top 20 cited journals with the strongest citation bursts.

After excluding peripheral nodes, a total of 1,041 journals were included in the journal network visualization analysis (Figure 6A). To improve the readability of the network map and highlight journals with relatively high academic influence in this field, a minimum co-citation threshold of 200 citations was applied, based on commonly adopted approaches in previous high-quality bibliometric studies and the citation distribution characteristics of the present dataset. Accordingly, only journals with ≥200 citations were displayed in the visualization map. This threshold helped preserve the major scholarly communication structure while reducing interference from low-frequency nodes. The top three journals in terms of publication output were Frontiers in Immunology (666 publications), The Journal of Immunology (183 publications), and PLOS ONE (139 publications) (Table 2). Journal co-citation analysis demonstrated that the most frequently co-cited journals were The Journal of Immunology (2,935 citations), Journal of Experimental Medicine (2,283 citations), and Proceedings of the National Academy of Sciences of the United States of America (2,077 citations), indicating their substantial academic influence in research related to Tregs and lung diseases. Journal citation burst analysis further revealed that International Journal of Molecular Sciences, Cells, and Frontiers in Immunology exhibited relatively strong citation burst intensities (Figure 6B), suggesting increased scholarly attention toward studies published in these journals in recent years.

**Table 2:** Top 10 journals and co-cited journals on research of Tregs in lung diseases.

Rank	Journals	Count	Co-cited journals	Citation
1	FRONT IMMUNOL	666	J IMMUNOL	2,935
2	J IMMUNOL	183	J EXP MED	2,283
3	PLOS ONE	139	P NATL ACAD SCI USA	2077
4	FRONT ONCOLOGY	125	J CLIN INVEST	1948
5	INT IMMUNOPHARMACOL	101	NATURE	1858
6	INT J MOL SCI	81	IMMUNITY	1853
7	CELLS	75	PLOS ONE	1831
8	CANCERS	74	NAT IMMUNOL	1773
9	J ALLERGY CLIN IMMUNOL	69	NAT REV IMMUNOL	1731
10	J IMMUNOTHER CANCER	64	AM J RESP CRIT CARE	1,677

### 3.6 Keywords

**Figure 7:**
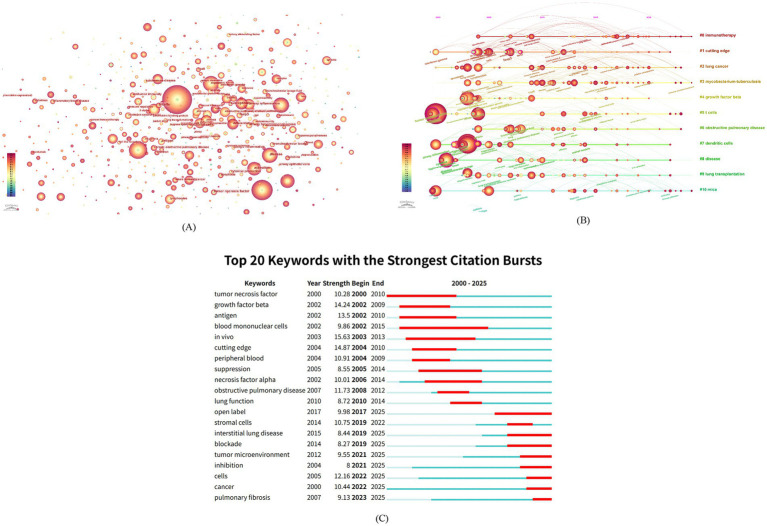
**(A)** Visualization map of keywords analysis. **(B)** Timeline view of keywords clustering analysis. **(C)** Top 20 keywords with the strongest citation bursts.

In the keyword co-occurrence analysis, a total of 692 keywords were included in the visualization after excluding low-frequency nodes (Figure 7A). Keyword frequency analysis showed that the top 10 most frequently occurring keywords were “*regulatory T cells*,” “*expression*,” “*dendritic cells*,” “*inflammation*,” “*disease*,” “*lung cancer*,” “*activation*,” “*responses*,” “*mice*,” and “*airway inflammation*” (Table 3). The top 10 keywords ranked by centrality included “*B cells*,” “*antigen*,” “*antitumor immunity*,” “*cancer immunotherapy*,” “*autoimmune diseases*,” “*CD4 + CD25 + T cells*,” “*chemoattractant cytokine*,” “*T cells*,” “*atopic asthma*,” and “*deficient mice*,” indicating that these keywords played important bridging roles among different research topics. Further keyword clustering analysis using CiteSpace generated 11 major clusters (Figure 7B), including “#0 *immunotherapy*,” “#2 *lung cancer*,” “#3 *mycobacterium-tuberculosis*,” “#4 *growth factor beta*,” “#6 *obstructive pulmonary disease*,” and “#9 *lung transplantation*.” Among them, “#0 *immunotherapy*” represented the largest keyword cluster and remained active in the timeline view until recent years. Clusters such as “#2 *lung cancer*” and “#6 *obstructive pulmonary disease*” persisted across multiple time periods, reflecting sustained attention to these topics throughout different stages of research development.

**Table 3:** Top 10 documents with the most citations.

Rank	Tittle	References	Citations
1	Mechanisms of fibrosis: therapeutic translation for fibrotic disease	Wynn TA, 2012, NAT MED, V18, P1028, DOI 10.1038/nm.2807	2,849
2	The development of allergic inflammation	Galli SJ, 2008, NATURE, V454, P445, DOI 10.1038/nature07204	1,462
3	Fibrotic disease and the TH1/TH2 paradigm	Wynn TA, 2004, NAT REV IMMUNOL, V583, P94, DOI 10.1038/nri1412	1,399
4	Targeting the TGFβ signalling pathway in disease	Akhurst RJ, 2012, NAT REV DRUG DISCOV, V790, P811, DOI 10.1038/nrd3810	1,255
5	Emerging pathogenic links between microbiota and the gut-lung axis	Budden KF, 2017, NAT REV MICROBIOL, V15, P55, DOI 10.1038/nrmicro.2016.142	1,155
6	Immunology of asthma and chronic obstructive pulmonary disease	Barnes PJ, 2008, NAT REV IMMUNOL, V8, P183, DOI 10.1038/nri2254	1,080
7	TGF-β and the TGF-*β* Family: Context-Dependent Roles in Cell and Tissue Physiology	Morikawa M, 2016, COLD SPRING HARB, DOI 10.1101/cshperspect.a021873	1,055
8	Anti-PD-1 and Anti-CTLA-4 Therapies in Cancer: Mechanisms of Action, efficacy, and Limitations	Seidel JA, 2018, FRONT ONCOL, V8, P86, DOI 10.3389/fonc.2018.00086	947
9	Immune cell promotion of metastasis	Kitamura T, 2015, NAT REV IMMUNOL, V73, P86, DOI 10.1038/nri3789	890
10	Clinical Impact of Different Classes of Infiltrating T Cytotoxic and Helper Cells (Th1, Th2, Treg, Th17) in Patients with Colorectal Cancer	Tosolini M, 2011, CANCER RES, V1263, P71, DOI 10.1158/0008-5472. CAN-10-2907	899

Keyword burst analysis further illustrated the temporal evolution of research hotspots in this field (Figure 7C). During the early developmental stage (2000–2010) keywords with the strongest burst intensities mainly included “tumor necrosis factor” (strength = 10.28) “growth factor beta” (strength = 14.24)“antigen” (strength = 13.85) “*in vivo*” (strength = 15.63)and “cutting edge” (strength = 14.87). These findings indicate that early investigations primarily focused on fundamental immune regulatory mechanisms cytokine-mediated inflammatory responses and experimental immunology associated with Treg biology. After 2010 the research focus gradually expanded toward pulmonary inflammatory disorders and translational immunology. Among these topics “obstructive pulmonary disease” exhibited a notable citation burst during 2008–2012 while “lung function” remained an active keyword from 2010 to 2014 reflecting increasing attention toward the immunopathological mechanisms underlying chronic airway diseases and pulmonary functional impairment. In addition keywords such as “suppression,” “necrosis factor alpha,” and “peripheral blood” further suggested continued interest in systemic immune regulation and inflammatory signaling pathways during this period.

In recent years, emerging burst keywords increasingly reflected the rapid development of tumor immunology and chronic pulmonary disease research. Keywords such as “open label” (2017–2025), “blockade” (2019–2025), “inhibition” (2021–2025), “tumor microenvironment” (2021–2025), and “cancer” (2022–2025) showed sustained citation bursts, indicating growing interest in immune checkpoint blockade and tumor-related immunotherapy. Meanwhile, “interstitial lung disease” (2019–2025) and “pulmonary fibrosis” (2023–2025) also emerged as recent burst keywords, suggesting increasing attention toward Treg-mediated immune regulation in chronic fibrotic lung diseases. Overall, these findings indicate that current research hotspots have gradually shifted from basic immune regulation toward translational studies involving tumor immunology, pulmonary fibrosis, and microenvironment-related immune responses.

### 3.7 Citation and co-citation analysis

**Figure 8:**
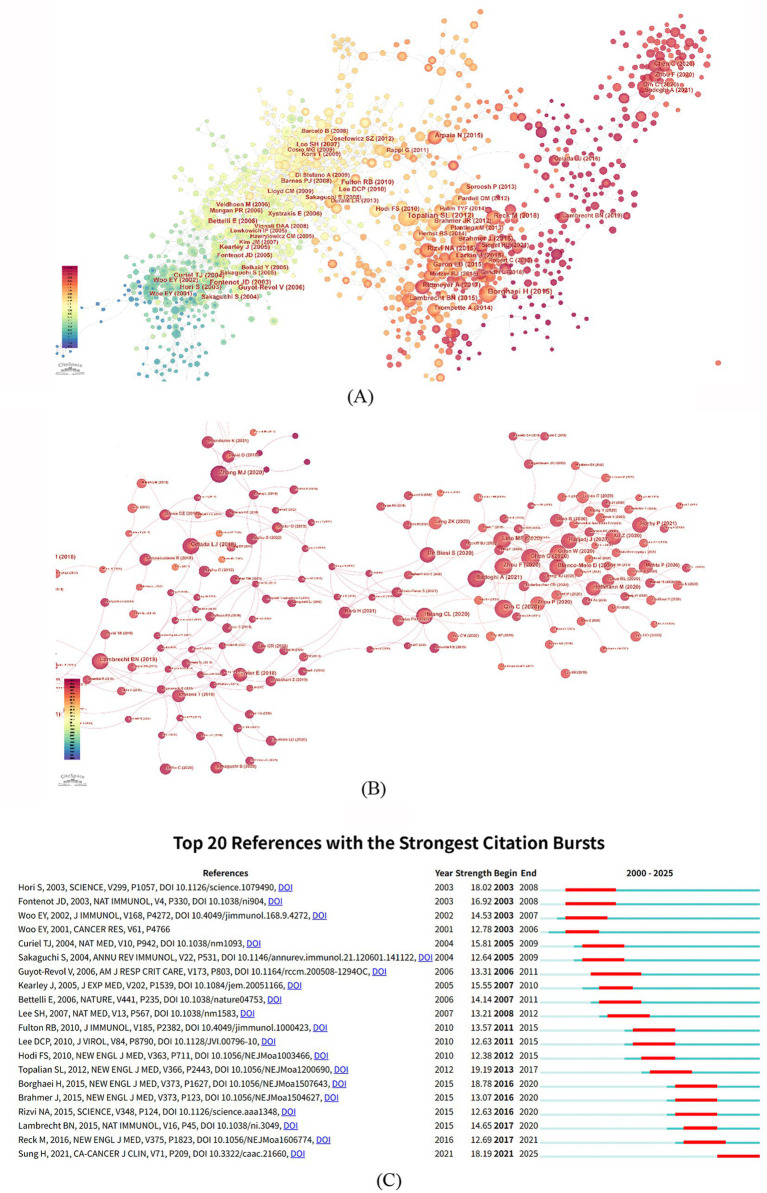
**(A)** Visualization map of references analysis. **(B)** Visualization map of references analysis over the last 10 years. **(C)** Top 20 references with the strongest citation bursts.

Citation frequency of references can reflect the core knowledge base and academic influence within a specific research field (Figures 8A,B). In the present study, co-citation analysis was performed using CiteSpace, and the top 10 most frequently cited references were identified, with citation counts ranging from 899 to 2,849 (Table 3). Highly cited references were mainly associated with pulmonary inflammation, fibrosis, tumor immunology, and immune regulation. Among these, the most highly cited reference was *Mechanisms of Fibrosis: Therapeutic Translation for Fibrotic Disease*, which primarily focused on immune regulatory mechanisms involved in fibrosis. This study reported that TGF-*β*1 secreted by Tregs may exert anti-inflammatory and antifibrotic effects. The second-ranked reference, *The Development of Allergic Inflammation*, focused on immune cell regulation during allergic inflammation and likewise discussed the capacity of Tregs to produce mediators, cytokines, chemokines, and growth factors involved in inflammation attenuation and tissue repair. Studies directly related to TGF-β signaling in Tregs also exhibited high citation frequencies, including *Targeting the TGFβ signalling Pathway in Disease* and *TGF-β and the TGF-β Family: Context-Dependent Roles in Cell and Tissue Physiology*. These studies were among the highly cited references because of the broad immunoregulatory functions of TGF-β and their discussion of TGF-β secretion by Tregs. In addition, references such as *Emerging Pathogenic Links between Microbiota and the Gut-Lung Axis* addressed the relationship between the gut–lung axis and immune regulation in lung diseases, and reported that certain gastrointestinal Clostridium species may promote anti-inflammatory Treg responses and suppress allergic airway inflammation.

The timeline view demonstrated that highly co-cited references formed a relatively continuous evolutionary trajectory across different research stages (Figure 8A). Early highly co-cited studies mainly focused on the fundamental immunoregulatory functions of Tregs and their roles in inflammatory responses, including *Control of Regulatory T Cell Development by the Transcription Factor Foxp3* and *Cutting Edge: Regulatory T Cells Suppress Cytokine Production by Dendritic Cells*. Subsequently, co-cited studies gradually expanded toward asthma, chronic obstructive pulmonary disease, pulmonary fibrosis, and tumor immunology, including *Immunology of Asthma and Chronic Obstructive Pulmonary Disease*, *Mechanisms of Fibrosis: Therapeutic Translation for Fibrotic Disease*, and *Anti–PD-1 and Anti–CTLA-4 Therapies in Cancer*, which involve targeted therapies related to Tregs intervention.

Reference burst analysis revealed that references with strong citation bursts were predominantly related to immune checkpoint inhibitors and cancer immunotherapy research (Figure 8C). Among them, *Nivolumab versus Docetaxel in Advanced Squamous-Cell Non–Small-Cell Lung Cancer*, *Pembrolizumab versus Docetaxel for Previously Treated, PD-L1-Positive, Advanced Non–Small-Cell Lung Cancer(KEYNOTE-010): A Randomised Controlled Trial*, and *Nivolumab in Previously Untreated Melanoma without BRAF Mutation* exhibited sustained citation bursts during 2016–2021. These studies mainly involved PD-1/PD-L1-related immunoregulatory pathways in Tregs and the clinical application of immunotherapy. In addition, recent burst references also included studies associated with the tumor microenvironment, inflammatory immune regulation, and chronic inflammatory lung diseases, such as *The Microbiome and Lung Cancer*, suggesting sustained scholarly attention to these research directions in recent years.

## 4 Discussion

### 4.1 General distribution and collaborative landscape

The annual publication trend showed a continuous increase in scholarly attention (Figure 2), reflecting both growing scientific interest and the expanding clinical relevance of Treg studies in pulmonary diseases. The United States and China were the leading contributors in publication output. While the United States exhibited higher centrality, indicating its pivotal role in international collaboration and knowledge dissemination (Figures 3A,B), China has shown rapid growth in recent years, though its relatively lower centrality suggests that broader international collaboration could further enhance its global academic influence.

At the institutional level, Harvard Medical School, the Ministry of Education of China, INSERM, and the NIH were among the most productive organizations, while Brigham & Women’s Hospital, the University System of Ohio, Johns Hopkins Medicine, and the NIH showed high centrality, highlighting their bridging roles in international collaboration (Figure 4; Table 1). At the author level, Bart N Lambrecht had the highest publication output, Philip M Hansbro exhibited the highest centrality, Shimon Sakaguchi was the most frequently co-cited author, and Peter J Barnes showed the strongest citation burst intensity (Figures 5A,B; Table 4). Journal distribution further reflected the interdisciplinary nature of the field, with major publications concentrated in immunology, respiratory medicine, oncology, and translational research journals (Table 2; Figure 6).

**Table 4:** Top 10 authors and co-cited authors on research of Tregs in lung diseases.

Rank	Authors	Count	Co-cited authors	Citation
1	Lambrecht, B. N.	24	Sakaguchi S.	482
2	Finotto, S.	22	Fontenot J. D.	235
3	Blazar, B. R.	20	Barnes, Peter J.	226
4	Loures, F. V.	20	Hori S.	206
5	Calich, V. L. G.	19	Wang J.	173
6	Taube, C.	18	Zhang Y.	171
7	Gelfand, E. W.	16	Lambrecht B. N.	160
8	Chen, J.	15	Miyara M.	156
9	Grunewald, J.	15	Bettelli E.	140
10	Hammad, H.	15	Shevach E. M.	135

Collectively, these patterns provide a concise view of the structural and quantitative landscape of Treg-related pulmonary disease research, highlighting both established contributors and emerging global trends while linking institutional and author activity with knowledge dissemination across countries and disciplines.

### 4.2 Hotspots and frontiers identified by bibliometric analysis

Bibliometric analyses, including keyword co-occurrence, clustering, citation burst, and co-citation networks, revealed the main research themes and emerging trends in Treg-related pulmonary disease studies. The results suggest that the field has gradually shifted from foundational studies on immune regulation toward more translational and clinically oriented topics. Major hotspots identified include immune regulation and inflammatory homeostasis, tumor immunology and immune checkpoint blockade, microbiota-associated immune modulation, and interstitial lung disease or fibrotic remodeling (Figures 7, 8; Table 5). These themes provide a framework for understanding both long-standing research focus and emerging frontiers.

**Table 5:** Top 10 keywords on research of Tregs in lung diseases.

Rank	Keyword	Count	Keywords	Centrality
1	regulatory T cells	1,659	B cells	1.19
2	expression	655	antigen	1.13
3	dendritic cells	485	antitumor immunity	1.11
4	inflammation	382	cancer immunotherapy	1.03
5	disease	353	autoimmune diseases	1.01
6	lung cancer	298	CD4^+^CD25^+^ T cells	0.99
7	activation	294	chemoattractant cytokine	0.96
8	responses	218	T cells	0.85
9	mice	213	atopic asthma	0.83
10	airway inflammation	199	deficient mice	0.78

#### 4.2.1 Immune regulation and inflammatory homeostasis

Immune regulation remained a foundational hotspot in Treg-related pulmonary disease research. In our keyword co-occurrence and burst analyses, high-frequency terms such as “regulatory T cells,” “inflammation,” “activation,” “responses,” and “airway inflammation” were prominent, while early burst keywords including “antigen,” “tumor necrosis factor,” and “growth factor beta” suggested that early studies focused on antigen-driven immune responses, cytokine regulation, and inflammatory signaling pathways (Figure 7A; Table 5). Co-citation and burst analyses further highlighted highly influential references associated with allergic inflammation, fibrosis, cytokine networks, and TGF-*β*-related immune regulation. Notably, *Mechanisms of Fibrosis: Therapeutic Translation for Fibrotic Disease* and *The Development of Allergic Inflammation* were repeatedly co-cited, reflecting that studies addressing inflammatory regulation and tissue remodeling have formed a structurally central and enduring knowledge base within pulmonary Treg research (10, 11). Together, these bibliometric patterns demonstrate that immune regulation and inflammatory homeostasis are consistently recognized as key themes across the literature.

From a biological perspective, the prominence of these topics aligns with evidence that Tregs suppress excessive inflammatory responses, maintain tolerance to inhaled antigens, and modulate interactions among epithelial cells, dendritic cells, macrophages, and effector T cells within the respiratory microenvironment (12–14). Lung-resident Tregs also contribute to tissue repair and regeneration during acute and chronic lung injury (15–17). In particular, TGF-β promotes Foxp3 expression and peripheral Treg stability, while participating in fibroblast activation, extracellular matrix deposition, airway remodeling, and tumor immune escape (18–20). These biological insights provide a mechanistic explanation for why Treg- and TGF-β-related topics are consistently prominent in the bibliometric analysis.

#### 4.2.2 Tumor immunology and immune checkpoint blockade

Tumor immunology and immune checkpoint blockade emerged as a major translational frontier in Treg pulmonary disease research. In keyword clustering analysis, “immunotherapy” represented the largest cluster, while “lung cancer” appeared as a high-frequency disease-related term (Table 5; Figure 7B). Citation burst analysis further highlighted terms such as “PD-1 blockade,” “nivolumab,” “blockade,” “tumor microenvironment,” and “cancer,” indicating that immune checkpoint therapy and tumor immune regulation have received increasing attention in recent years (Figure 7C). Reference co-citation and burst analyses identified highly influential studies published after 2015, including *Nivolumab versus Docetaxel in Advanced Squamous-Cell Non–Small-Cell Lung Cancer* and *Pembrolizumab versus Docetaxel for Previously Treated, PD-L1-Positive, Advanced Non–Small-Cell Lung Cancer* (21, 22). The repeated appearance of these studies in the co-citation network suggests that immune checkpoint inhibitors have strongly influenced the knowledge structure and research direction of pulmonary Treg studies (Figures 8A–C).

From a biological perspective, the prominence of tumor immunology-related topics can be explained by the role of Tregs in modulating antitumor immunity. Tumor-infiltrating Tregs can suppress cytotoxic CD8 + T-cell activity and facilitate immune evasion through pathways including IL-10, TGF-*β*, CTLA-4, and PD-1 signaling (23–25). The increasing emphasis on the “tumor microenvironment” in bibliometric patterns suggests that recent studies are extending beyond isolated Treg function toward multicellular immune interactions in lung cancer (26). These observations imply that future research may focus on how Treg-mediated immunosuppression affects checkpoint inhibitor efficacy, the use of Treg-related biomarkers for patient stratification, and strategies to selectively modulate Tregs to enhance antitumor responses while minimizing immune-related adverse events.

#### 4.2.3 Gut microbiota and the gut–lung axis

Microbiota-associated immune modulation has emerged as a relatively recent and specialized frontier in Treg pulmonary disease research. Although the gut–lung axis did not appear as a high-frequency keyword in our current dataset, bibliometric analysis of co-citation and citation bursts revealed influential references highlighting this concept, such as *Emerging Pathogenic Links Between Microbiota and the Gut-Lung Axis* (27). These findings suggest that while gut microbiota has not yet formed a dominant keyword cluster, it is increasingly recognized as an important factor in shaping systemic immune responses relevant to pulmonary Treg research.

From a biological perspective, intestinal microbial communities can influence lung immune homeostasis through mechanisms such as tolerogenic dendritic cell-mediated Treg differentiation and modulation of Th2/Th17 responses (28). The prominence of microbiota-related high-impact studies in co-citation networks reflects the growing appreciation that systemic immunoregulation, including gut–lung interactions, contributes to pulmonary disease outcomes. This emerging frontier points toward future research integrating microbiome profiling, immune phenotyping, and translational interventions (29).

#### 4.2.4 Interstitial lung disease and chronic inflammatory lung disorders

Interstitial lung disease (ILD) and fibrosis appeared as an independent research hotspot in bibliometric analyses, reflected by keyword bursts and co-citation clusters specifically associated with chronic lung injury and tissue remodeling (Figure 7C; Table 3). Although TGF-*β*-mediated signaling was already highlighted in 4.2.1 under immune regulation, the ILD/fibrosis cluster is distinguished in the bibliometric results because it emphasizes clinical and translational studies on chronic fibrotic remodeling, which constitute a separate emerging focus from foundational immune regulation.

Highly cited references in this cluster focus on fibrotic mechanisms, fibroblast activation, and extracellular matrix deposition (30). These bibliometric patterns indicate sustained attention to Treg-mediated regulation in the context of tissue repair and fibrosis. Biologically, Tregs contribute to controlling excessive inflammation and promoting tissue repair during chronic lung injury, including modulation of fibroblast activity, extracellular matrix deposition, and airway remodeling (31). The recent emergence of ILD-related keywords may also reflect heightened interest following COVID-19, where immune dysregulation and post-inflammatory pulmonary fibrosis became important clinical concerns (32). This distinct bibliometric signal justifies its separate discussion as an emerging translational frontier, highlighting the need for further mechanistic and clinical studies in fibrotic lung disorders.

### 4.3 Distinguishing bibliometric findings from biological interpretation

Bibliometric analysis provides a quantitative approach to mapping the knowledge structure, research hotspots, and emerging trends of a scientific field. In the present study, keyword co-occurrence, clustering, citation burst, and co-citation analyses identified several major themes in Treg-related pulmonary disease research, including immune regulation, tumor immunology, microbiota-associated immune modulation, and interstitial lung disease. These findings reflect the topics that have received sustained attention or have recently emerged in the literature, rather than direct experimental evidence for specific biological mechanisms.

Therefore, the biological interpretations discussed above should be understood as contextual explanations of the bibliometric patterns, supported by previous experimental and clinical studies. For example, the prominence of inflammation- and TGF-*β*-related terms suggests that cytokine-mediated immune regulation has remained a central topic in the literature, while mechanistic studies provide evidence for the roles of Tregs and TGF-β in immune tolerance, tissue repair, and fibrotic remodeling. Similarly, the emergence of immune checkpoint blockade and tumor microenvironment-related terms reflects a growing translational focus, whereas the underlying mechanisms require validation through experimental studies, clinical cohorts, and interventional trials. This distinction is important for interpreting bibliometric results appropriately and for guiding future research based on identified trends.

### 4.4 Comparison with previous bibliometric studies and unique contributions of the present study

Several previous bibliometric analyses have investigated immune regulation, cancer immunotherapy, microbiota research, or Treg-associated diseases independently (33, 34). Existing studies have mainly focused on Tregs in cancer immunotherapy, autoimmune diseases, or general immunology fields, while bibliometric investigations specifically targeting pulmonary diseases remain limited. In addition, prior pulmonary immunology bibliometric studies often concentrated on individual diseases such as asthma, COPD, or lung cancer rather than examining Treg-centered immune regulation across multiple pulmonary disorders.

Compared with these previous studies, the present analysis provides several novel contributions. First, this study systematically integrated diverse pulmonary diseases into a unified Treg-centered bibliometric framework, thereby revealing the interdisciplinary knowledge structure connecting pulmonary inflammation, fibrosis, tumor immunology, microbiota research, and immune checkpoint therapy. Second, by combining co-citation, burst detection, and keyword clustering analyses, the study identified a clear temporal evolution from foundational immune regulation toward translational immunotherapy and microbiome-associated research. Third, the present study highlighted emerging translational hotspots, including gut microbiota, interstitial lung disease, tumor microenvironment regulation, and PD-1/PD-L1 blockade, which may not have been comprehensively captured in earlier bibliometric analyses.

### 4.5 Limitations

Several limitations should be acknowledged in this study. First, the analysis was limited to publications indexed in the Web of Science Core Collection and Scopus databases, which may have excluded relevant studies from other databases. Second, only English-language articles and reviews were included, potentially introducing language bias. Third, bibliometric analyses are influenced by database updates and citation accumulation over time, meaning that recently published high-quality studies may not yet have achieved sufficient citation frequency. Fourth, although CiteSpace provides valuable visualization and network analyses, the interpretation of clustering results may still contain a degree of subjectivity. Despite these limitations, the present study comprehensively characterized the global research landscape of Tregs in pulmonary diseases and provided important insights into the field’s developmental trajectory and emerging frontiers.

## 5 Conclusion

In summary, this bibliometric study systematically analyzed global research trends, collaboration networks, knowledge structures, and emerging hotspots related to Tregs in pulmonary diseases from 2000 to 2025. The findings suggest that the field has gradually evolved from studies of immune tolerance and cytokine regulation toward translational research involving tumor immunotherapy, immune checkpoint blockade, gut microbiota, and chronic inflammatory lung diseases. Keyword and co-citation analyses identified lung cancer, tumor microenvironment, interstitial lung disease, and microbiota-associated immune regulation as major emerging frontiers, while TGF-*β*-related immune regulation remained a central and recurrent theme throughout the literature. Overall, this study provides a comprehensive overview of the development of Treg-related pulmonary research and may offer valuable insights for guiding future basic, translational, and clinical investigations in pulmonary immunology.

## Data Availability

Publicly available datasets were analyzed in this study. This data can be found at: Web of Science™ Core Collection, Scopus.
