# Supplementary material for: The evolving role of regulatory T cells in pulmonary diseases: immunomodulatory mechanisms and translational directions revealed by bibliometric analysis
Source: Front Med (Lausanne). 2026 Jun 9;13:1839305. doi: 10.3389/fmed.2026.1839305 (PMC13286889; doi:10.3389/fmed.2026.1839305)
Supplement: Supplementary file 1 [file Table_1.docx]

Supplementary Material 1

**WoSCC:**

TS = (("regulatory T cell*" OR "Treg*" OR "regulatory T lymphocyte*" OR "Foxp3+ T cell*" OR "CD4+CD25+Foxp3+") AND ("lung disease*" OR "pulmonary disease*" OR "respiratory disease*" OR "lung disorder*" OR "pulmonary disorder*" OR "lung injury*" OR "pulmonary fibrosis" OR "asthma" OR "COPD" OR "lung cancer"))

**Scopus:**

TITLE-ABS-KEY (("regulatory T cell*" OR "Treg*" OR "regulatory T lymphocyte*" OR "Foxp3+ T cell*" OR "CD4+CD25+Foxp3+") AND ("lung disease*" OR "pulmonary disease*" OR "respiratory disease*" OR "lung disorder*" OR "pulmonary disorder*" OR "lung injury*" OR "pulmonary fibrosis" OR "asthma" OR "COPD" OR "lung cancer"))
